# Acute sensorineural hearing loss and severe otalgia due to scrub typhus

**DOI:** 10.1186/1471-2334-9-173

**Published:** 2009-10-22

**Authors:** Ji-In Kang, Dong-Min Kim, Joonhan Lee

**Affiliations:** 1 Division of Infectious Diseases, Department of Internal Medicine, Chosun University, School of Medicine, Gwangju City, Republic of Korea; 2 Department of Otorhinolaryngology - Head and Neck Surgery, Chosun University, School of Medicine, Gwangju City, Republic of Korea; 3 Research Center for Resistant Cell, Chosun University, School of Medicine, Gwangju City, Republic of Korea

## Abstract

**Background:**

Scrub typhus is an acute febrile illness caused by *Orientia tsutsugamushi*.

**Case presentations:**

We encountered a patient with sensorineural hearing loss complicating scrub typhus, and three patients with scrub typhus who complained of otalgia, which was sudden onset, severe, paroxysmal, intermittent yet persistent pain lasting for several seconds, appeared within 1 week after the onset of fever and rash. The acute sensorineural hearing loss and otalgia were resolved after antibiotic administration.

**Conclusion:**

When patients in endemic areas present with fever and rash and have sensorineural hearing loss or otalgia without otoscopic abnormalities, clinicians should suspect scrub typhus and consider empirical antibiotic therapy.

## Background

Scrub typhus is an acute febrile illness caused by *Orientia tsutsugamushi*, an obligate intracellular bacterium; it is transmitted via the bites of *Leptotrombidium *chigger mites [1]. *O. tsutsugamushi *spreads throughout the body via the blood and lymphatic vessels, so that patients infected with this microorganism manifest a variety of clinical symptoms and signs such as myalgia and diffuse lymphadenopathy [2]. Complications include pneumonia, myocarditis, meningitis, hepatitis, acute renal failure and hearing loss [3-5]. A sensory neural type of hearing loss has been reported in patients infected with *Rickettsia rickettsii *(*R. rickettsii), R. typhi *or *R. coronii *[6-8]. Hearing loss described in scrub typhus infected patients is rare in Korea. However, in a recent Sri Lanka study, hearing loss appeared in 19% of patients with scrub typhus, and can affect up to one third of the patients [3,4]. However, the performance of audiometry studies in these patients is not common.

We report four confirmed cases of scrub typhus with otological complaints. One patient presented with sensory-neural hearing loss and other three patients presented with severe otalgia.

## Case Presentation

**Figure 1:**
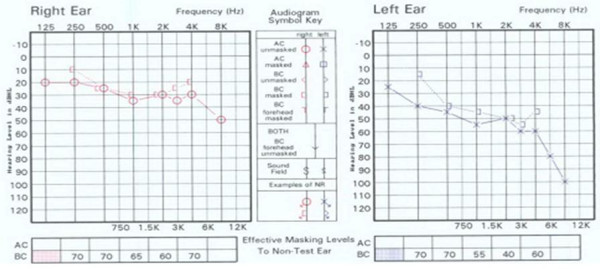
**Pure tone audiograms of the 59-year-old patient with scrub typhus and associated hearing impairment**. The hearing threshold level was 31 dB in the right ear and 52 dB in the left ear.

A 60-year-old woman was admitted to Chosun University Hospital with a one-week history of multiple erythematous rash on the face and whole body. There was a history of exposure to mites, about two weeks prior to presentation, during a visit to the grave of one of her ancestor. Two days later, generalized maculopapular rash appeared with fever. From approximately the 10th day after the onset of fever and rash, she had hearing loss without tinnitus, and could not hear the sounds on television. She was transferred to Chosun University Hospital from a regional hospital. Her past medical history was not remarkable and she denied any recent administration of salicylates or aminoglycosides. She did not have any upper respiratory infections, otitis media or ear trauma. On admission, she was in an acute distressed state with body temperature of 39°C, pulse rate of 117 beats/min, respiratory rate of 24/min and blood pressure of 90/60 mm Hg. She was dehydrated, had conjunctival injection and her lymph nodes in the left inguinal region were tender. A 1 × 1-cm eschar was noted on the right flank area. Because of the presence of rash and eschar during an endemic season, she was empirically treated with rifampin (600 mg). Hematologic tests revealed normal leukocyte count and hemoglobin, and low platelet count (hemoglobin, 12.7 g/dL; WBC, 6420/mm^3^; platelets, 120,000/mm^3^). Biochemical tests were normal except for increased serum levels of aminotransferases (AST, 214 IU/L; ALT, 199 IU/L), and her urinalysis was normal. Serum C-reactive protein was 5.5 mg/L, erythrocyte sedimentation rate was 51 mm, blood urea nitrogen was 20.7 mg/dL and serum creatinine was 0.93 mg/dL. Peripheral blood smears for malaria and parasites, and blood and urine cultures, were all negative. Chest X-rays were normal. Indirect immunofluorescence assays revealed an IgM titer of 1:160 against *O. tsutsugamushi *on admission, and an IgM titer of 1:320 against *O. tsutsugamushi *at 2-week follow-up. The initial IgG titer of 1:1,024 rose to 1:4,096 two weeks later (Table 1). We performed PCR on the eschar and confirmed presence of the gene encoding a 56-kDa protein that is specific for *O. tsutsugamushi*. At 1-week follow up, pure tone audiometry performed in the outpatient clinic revealed a hearing threshold of 52.5 dB in the left ear, which was of the sensorineural type (Figure 1).

**Table 1:** Clinical and serologic findings in four patients with scrub typhus and otologic problems

**Patient No**.	Age/Sex	Eschar	Otologic problems			Serum antibody titer				PCR genotype
			Type	Days from symptom onset to otologic problems	Pure tone audiometry	Acute phase		Convalescent phase		
						IgM	IgG	IgM	IgG	
1	59/F	+	deaf	10	SNHL	160	1024	320	4096	Boryung

2	39/F	+	otalgia	2	Normal	40	32	320	256	Boryung

3	73/F	+	otalgia	4	ND	20	32	80	128	Taguchi

4	63/M	+	otalgia	1	ND	0	64	0	2048	Boryung

SNHL = sensorineural hearing loss, ND = not done

Patients 2-4 presented with unilateral otalgia associated with fever and rash. On presentation, generalized rash with eschars were observed, and a history of working in fields in recent past was obtained. Patient 2 & 3 presented with otalgia without any hearing loss. Otalgia was paroxysmal, intermittent and severe which was followed by persistent pain for several hours. Patient 4 had history of wearing hearing aids for two months before presenting to the hostpital with pain in the ear, and had no vertigo or tinnitus on admission. The otolaryngologic examination of patient 1, 2 & 3 was normal. Pure tone audiometery was normal in patient 2 and it could not be done in patient 3 & 4. All patients received antibiotic therapy under a clinical diagnosis of scrub typhus, and the otalgia was resolved within 1 week. A four-fold or greater increase in antibody titer against *O. tsutsugamushi *was observed in indirect fluorescence antibody assays in the convalescent stage compared to that in the acute stage.

## Discussion

The 56-kDa type-specific antigen is a major outer membrane protein located on the surface of *Orientia *species that may be involved in penetration into host cells [9]. This 56-kDa antigen is an immunodominant antigen that induces strong humoral immunity. It also contains both group-specific and type-specific epitopes, which are useful for the diagnosis of scrub typhus [9]. Nested PCR was performed with blood buffy coat or eschars and IFA was conducted with serum by the method described previously [10]. Primers 34 (5-TCA AGC TTA TTG CTA GTG CAA TGT CTGC-3) and 55 (5-AGG GAT CCC TGC TGC TGT GCT TGC TGCG-3) were used in the first PCR. Nested PCR primers 10 (5-GAT CAA GCT TCC TCA GCC TAC TAT AAT GCC-3) and 11 (5-CTA GGG ATC CCG ACA GAT GCA CTA TTA GGC-3) were used in the second PCR amplification of the resulting 483 bp fragment (Figure 1). The DNA sequences of amplicons were compared with the nucleotide sequences of *O. tsutsuganushi *registered in GenBank.

Our patients presented with scrub typhus and associated sensory neural hearing loss and severe otalgia. A sensory neural type of hearing loss has been reported in patients infected with *Rickettsia rickettsii *(*R. rickettsii), R. typhi *or *R. coronii *[6-8]. Although the mechanism of hearing loss in scrub typhus has not yet been elucidated, at least two mechanisms have been proposed. In the first, the rickettsiae directly invade the central nervous system and induce vasculitis in the acute stage, and this damages the cochlear division of the eighth cranial nerve [6,11,12]. In the second, vasculitis is produced in the vasa vasorum of the cochlear nerve by a secondary immune mechanism. In our first patient, acute hearing loss was so severe that she could not hear the sounds on television; this was suggestive of bilateral sensorineural hearing loss. Pure tone audiometry performed after administration of rifampin revealed that her hearing had only improved in the right ear. Rifampin was administered based on a randomized trial by Watt et al reporting superior clinical results for treating scrub typhus patients with rifampin compared to doxycycline [13]. In this patient, hearing loss was of the sensory neural type, as it is in other rickettsial disease. Premarantna et al have reported that deafness and tinnitus appear on the second week after the onset of scrub typhus [3]. Similarly, in our patient sensorineural hearing loss appeared 10 days after the onset of scrub typhus. However, otalgia in three patients appeared within the first week. Thus, hearing loss and tinnitus tend to appear on the second week after the onset of scrub typhus, whereas otalgia appears in the first week. This may indicate that the mechanism of hearing loss and tinnitus may differ from that of otalgia. Further studies are needed to determine whether hearing loss, and otalgia are caused by direct damage to the sensory nerves or by a secondary immune mechanism. Although the definite pathology of sensorineural hearing loss is not known, such complications seem to respond to effective antibiotic treatment, early diagnosis of these complications and prompt institution of antirickettsial therapy are crucial for achieving good clinical outcomes.

## Conclusion

When patients in endemic areas present with fever and rash and have sensorineural hearing loss or severe otalgia without otoscopic abnormalities, clinicians should suspect scrub typhus and consider empirical antibiotic therapy.

## Consent

Written informed consent was obtained from the patient or their relative for publication of this study. A copy of the written consent is available for review by the Editor-in-Chief of this journal.

## Competing interests

The authors declare that they have no competing interests.

## Authors' contributions

JIK took care of the patients and drew up the first draft of the report, JHL, consultant otorhinolaryngologist, made a substantial contribution to draft the manuscript, and revised the draft all over the course of submission, DMK conceived of the study, participated in its design and coordination and drafted the manuscript. All authors read and approved the final manuscript.

## Pre-publication history

The pre-publication history for this paper can be accessed here:

http://www.biomedcentral.com/1471-2334/9/173/prepub
